# LncRNA VPS9D1-AS1 Promotes Malignant Progression of Lung Adenocarcinoma by Targeting miRNA-30a-5p/KIF11 Axis

**DOI:** 10.3389/fgene.2021.807628

**Published:** 2022-01-24

**Authors:** Jiefeng Liu, Yuhua Feng, Xinyu Zeng, Miao He, Yujing Gong, Yiping Liu

**Affiliations:** ^1^ Department of General Surgery, Changsha Hospital Affiliated to Hunan Normal University/the Fourth Hospital of Changsha, Changsha, China; ^2^ Department of Oncology, the Second Xiangya Hospital Central South University, Changsha, China; ^3^ Department of Oncology, Xiangya Hospital Central South University, Changsha, China

**Keywords:** VPS9D1-AS1, miRNA-30a-5p, KIF11, malignant progression, lung adenocarcinoma

## Abstract

**Objective:** This research probed into the molecular mechanisms of long non-coding RNA (lncRNA) VPS9D1 Antisense RNA 1 (VPS9D1-AS1) in lung adenocarcinoma (LUAD).

**Methods:** lncRNA expression level was evaluated bioinformatically, and its downstream miRNA/mRNA regulatory axis was predicted by bioinformatics methods as well. qRT-PCR was used to measure VPS9D1-AS1, miRNA-30a-5p, and kinesin family member 11 (KIF11) expression. Western blot was performed to measure KIF11 protein expression. Proliferation, migration, and invasion of LUAD cells were all observed by cell biological function experiments. Dual-luciferase assay detected binding between miRNA-30a-5p and VPS9D1-AS1 or KIF11, respectively. RIP experiment detected interaction between VPS9D1-AS1 and miRNA-30a-5p.

**Results:** VPS9D1-AS1 and KIF11 were increased in LUAD, whereas miRNA-30a-5p was decreased. VPS9D1-AS1 promoted the malignant progression of LUAD cells and could sponge miRNA-30a-5p. MiRNA-30a-5p could restore the impact of VPS9D1-AS1 on LUAD cells. KIF11 was a target downstream of miRNA-30a-5p. VPS9D1-AS1 could upregulate KIF11 expression through competitively sponging miRNA-30a-5p, and KIF11 could restore the impact of miRNA-30a-5p on LUAD cells.

**Conclusion:** VPS9D1-AS1 could foster malignant progression of LUAD *via* regulating miRNA-30a-5p/KIF11 axis, suggesting that VPS9D1-AS1 is key to regulating the malignant progression of LUAD.

## Introduction

Competing endogenous RNAs (ceRNAs) involved in post-transcriptional regulation (Salmena et al., 2011) play an important role in human physiological and pathological mechanisms. Considering the mechanisms of ceRNAs, long non-coding RNA (lncRNA)–miRNA-mRNA logic is prevalently studied. Briefly, the logic explained that lncRNA competingly sponges miRNA, which can regulate mRNA expression by targeting its 3′ untranslated region (3′UTR), resulting in disturbing suppressing effects of miRNA on mRNA expression (Bartel, 2009; Thomas et al., 2010; Wang et al., 2010). Recently, lncRNA-related ceRNA mechanisms have been considered as one crucial factor affecting tumor development.

lncRNA has a length of more than 200 nucleotides (Niu et al., 2017). At present, lncRNA is proved to exert a vital modulatory role in cell biological processes, including X chromosome imprinting, stem cell differentiation, immune response, and chemical resistance (Cai et al., 2019). Meanwhile, recent studies found that lncRNA is often dysregulated in lung adenocarcinoma (LUAD) and presents the function of regulating the progression of lung cancer. For example, Hongyue Zhang reported that HOXA-AS3 is upregulated in LUAD and can promote the proliferation and migration of LUAD cells (Liu et al., 2018). In another paper, Ming Zhao also reported that GMDS-AS1 is decreased in LUAD, inhibiting the proliferation of LUAD cells while promoting apoptosis of LUAD cells simultaneously (Zhao et al., 2020). However, far less has been understood lncRNA-related ceRNA mechanisms in LUAD.

In our study, VPS9D1 Antisense RNA 1 (VPS9D1-AS1) is comprehensively examined and analyzed to expand the understandings of it in LUAD. Several studies have implied its roles in prostate cancer so far. For example, it was reported that VPS9D1-AS1 can upregulate the expression of myocyte enhancer factor 2D through competitively sponging miRNA-4739, thus promoting the malignant progression of prostate cancer (Wang et al., 2020). Jinhua Wang also indicated that lncRNA VPS9D1-AS1 can promote the proliferation of prostate cancer *via* miRNA-184/c-Myc axis (Wang et al., 2018). Nevertheless, the specific regulatory mechanism of VPS9D1-AS1 in LUAD is warranted. Therefore, we confirmed that VPS9D1-AS1 could promote the malignant progression of LUAD through *in vitro* experiments. Moreover, the molecular mechanism of VPS9D1-AS1 was investigated in promoting malignant progression of LUAD. This work provides a theoretical basis for VPS9D1-AS1 to be a possible target for treating patients with LUAD.

## Materials and Methods

### Bioinformatics Analysis

The gene expression data of LUAD (59 normal samples and 535 cancer samples) and data of mature miRNAs (46 normal samples and 521 cancer samples) were downloaded from The Cancer Genome Atlas (TCGA) database (https://portal.gdc.cancer.gov/). On the basis of the downloaded data, a *t*-test was used to determine VPS9D1-AS1 level in normal and cancer tissues. Then, the cancer samples were classified into high- and low-expression groups on the basis of the median value of VPS9D1-AS1 expression. Survival analysis was done by R package “Survival” (R package version 3.6.1, Kaplan-Meier method). Then, subcellular localization analysis was performed on the target lncRNA *via* lncATLAS database (http://lncatlas.crg.eu/).

Differential analysis of miRNA (log |FC| > 1.5, FDR < 0.01) and mRNA (log |FC| > 2.0, FDR< 0.01) between normal group and tumor group was performed on the basis of gene expression data by using R package “EdgeR.” At the same time, LncBase database was used to predict miRNAs that had interaction with VPS9D1-AS1. The predicted miRNAs were overlapped with downregulated miRNAs. After Pearson correlation analysis, miRNA with the highest negative correlation was the downstream target regulated by VPS9D1-AS1. Four databases including miRDB, mirDIP, miRTarBase, and starBase were used to predict the downstream mRNAs of target miRNA. The predicted mRNAs were intersected with the upregulated mRNAs. The correlation between target mRNAs and miRNAs was calculated to determine the target mRNA. Survival analysis of the confirmed target mRNA was carried out. The correlation between the target mRNA and clinical features was determined by Wilcoxon test (2 groups) or Kruskal test (>2 groups).

### Cell Culture

Human normal lung epithelial cells BEAS-2B (3131C0001000200027), LUAD cells A549 (3131C0001000700150), Calu-3 (3131C0001000700157), H1975 (3131C0001000700193), and SPC-A-1 (3131C0001000700053) were all accessed from Cell Resource Centre of Shanghai Institutes for Biological Sciences, Chinese Academy of Sciences. All the above cells were maintained in DMEM (Thermo Fisher Scientific) containing 10% FBS (Thermo Fisher Scientific). The culture conditions in the incubator were 5% CO_2_ and 37°C in a humid environment.

### Cell Transfection

For overexpressing VPS9D1-AS1 in cells, VPS9D1-AS1 was amplified and cloned into pcDNA3.1 (Invitrogen, Carlsbad, USA). Then, 50 nM pcDNA3.1-LUCAT1 (oe-VPS9D1-AS1) or negative antibody [oe-negative control (NC)] was transfected into A549 cells by 6 μl of Lipofectamine 2000 (Invitrogen, Carlsbad, USA). The transfected cells were screened by puromycin (5 μg/ml; Sigma Aldrich, USA).

For silencing VPS9D1-AS1, overexpressing miRNA-30a-5p and overexpressed kinesin family member 11 (KIF11) cells: sh-VPS9D1-AS1, miRNA-30a-5p-mimic, and oe-KIF11, and their corresponding NCs were all purchased from GeneChem (Shanghai, China). The 50 nM sh-VPS9D1-AS1, miRNA-30a-5p-mimic, oe-KIF11, and their respective NCs were transfected into the cells by 6 μl of Lipofectamine 2000 (Invitrogen, USA), respectively. After 48 h of transfection, cells were cultured for 24 h under the condition of 37°C and 5% CO_2_ and collected for the subsequent experiment.

### qRT-PCR and Subcellular Fractionation

Total RNA was extracted with the RNeasy Mini Kit (Qiagen, New York, USA). lncRNAs and mRNAs were reversely transcribed with the PrimeScript RT Kit (Qiagen, New York, USA). miRNA was reversely transcribed with the Bulge-Loop miRNA qRT-PCR Starter Kit (RiboBio, Guangzhou, China). Then, qRT-PCR analysis was done on ABI PRISM 7900 Sequence Detection System (Applied Biosystems, USA) according to the manual of SYBR Green Master Mix (Applied Biosystems, USA). Both lncRNA and mRNA took GAPDH as internal reference, whereas miRNA took U6 as internal reference. Relative expressions of VPS9D1-AS1, miRNA-30a-5p, and KIF11 were obtained by 2^−ΔΔCT^ method. Primer sequences were shown in Table 1.

**TABLE 1:** qRT-PCR primer sequences.

Genes	Primer sequences
VPS9D1-AS1	Forward: 5′-CTC​AGC​AGT​AAG​TAA​CAG​TGG​TAG​A-3′
	Reverse: 5′-CTT​CAG​CAT​CTT​GGA​GGT​GTC-3′
miRNA-30a-5p	Forward: 5′-GGG​CCT​GTA​AAC​ATC​CTC​G-3′
	Reverse: 5′-GAA​TAC​CTC​GGA​CCC​TGC-3′
KIF11	Forward: 5′‐GCC​CCA​AAT​GTG​AAA​GCA​TT‐3′
	Reverse: 5′‐CTA​AAG​TGG​GCT​TTT​TGT​GAA​CTC​T‐3′
GAPDH	Forward: 5′-GGA​GCG​AGA​TCC​CTC​CAA​AAT-3′
	Reverse: 5′-GGC​TGT​TGT​CAT​ACT​TCT​CAT​GG-3′
U6	Forward: 5′-GGT​CGG​GCA​GGA​AAG​AGG​GC-3′
	Reverse: 5′-GCT​AAT​CTT​CTC​TGT​ATC​GTT​CC-3′

The cytoplasm and nucleus of A549 cells were isolated with the PARIS Kit (Thermo Fisher Scientific, USA). Then, RNA was separated from the two parts, with GAPDH as cytoplasmic control and U6 as nuclear control. Finally, qRT-PCR was used for analysis.

### Western Blot Assay

The transfected cells were cultured for 48 h and then collected for Western blot assay. The cells were lysed with radioimmunoprecipitation assay lysis buffer (Thermo Fisher Scientific) at 4°C for 10 min. Then, protein concentration was quantified by the BCA Protein Analysis Kit (Beyotime, China). SDS-PAGE was utilized to electrophorese equal amounts of protein samples. Then, proteins were transferred to a PVDF membrane. At room temperature, the membrane was sealed for 1 h with 5% skimmed milk diluted with TBST. Then, primary antibody KIF11 (Abcam) or GAPDH (Abcam) was added, and the membrane was incubated overnight at 4°C. The membrane was fully rinsed with TBST, followed by hybridizing with goat anti-rabbit IgG antibody conjugated with horseradish peroxidase (Abcam, Cambridge, UK) for 2 h. After that, TBST was utilized to rinse the membrane three times. Finally, luminescence was carried out with an enhanced chemiluminescence (ECL) kit (Solarbio, China). All protein bands were observed by photo taking.

### Cell Counting Kit-8 Assay

The transfected cells were uniformly suspended and inoculated into 96-well plates (2 × 10^3^ cells/well) and cultured at 37°C for 24 h. Zero, 24, 48, and 72 h later, 10 μl of CCK-8 solution (Dojindo Laboratories, Mashiki-machi, Japan) was added into wells, and the cells were incubated under routine conditions for 4 h. Then, the optical density (OD) value of each well at 450 nm was assayed by a microplate reader (Molecular Devices, Sunnyvale, CA, USA).

### Colony Formation Assay

The transfected cells were uniformly suspended and inoculated into six-well plates (4 × 10^2^ cells/well). The six-well plates were cultured in an incubator at 37°C with 5% CO_2_ for 24 h. After 2 weeks, the formed visible spots were confirmed with the naked eye. Then, 4% paraformaldehyde was utilized to fix cells for 15 min, and 0.1% crystal violet was utilized to stain the cells for 10 min. Phosphate-buffered saline (PBS) was utilized to rinse the excessive crystal violet dye in the wells. The number of colonies was counted.

### Scratch Healing Assay

The transfected cells were uniformly suspended and inoculated into six-well plates (8 × 10^5^ cells/well), and lines were drawn at the back side of plates with a marker pen. The six-well plates were cultured in an incubator at 37°C with 5% CO_2_ for 24 h. After 90% of the wells were covered with cells, a straight line was drawn with a 200-µl pipette tip, and the shedding cells in the wells were washed with PBS. After scratching for 0 and 24 h, the scratched area was photographed under inverted microscope (Zeiss, CFM-500, Oberkochen, Germany) with ×40 magnification. The cell migration rate was calculated. Cell migration rate = (scratch area at 0 h − scratch area at 24 h)/scratch area at 0 h. The assay was independently repeated three times (including three technical replicates and three biological replicates).

### Cell Invasion Assay

The transfected cells (8 × 10^5^ cells/well) were evenly suspended and planted into the upper Transwell chamber (Sigma, St. Louis, USA) containing Matrigel (BD Biosciences, USA). Afterward, 650 μl of DMEM plus 20% FBS was added to the lower Transwell chamber, and cells were maintained in an incubator for 24 h under routine conditions. After cell culture, cells were fixed with 4% paraformaldehyde for 15 min and stained with 0.1% crystal violet for 10 min. Cells in the upper insert were carefully swabbed off with a moist cotton swab. Five fields were randomly chosen to take pictures with an inverted microscope (Zeiss, CFM-500, Oberkochen, Germany) with ×40 magnification. The cells were counted.

### RNA Binding Protein Immunoprecipitation

RIP analysis was done with the EZ-Magna RIP Kit (Millipore, USA). In short, A549 and H1975 cells were lysed in RIP lysis buffer at 70%–80% fusion. Then, the magnetic beads were coupled with human anti-AGO2 antibody (Millipore) and normal humanized IgG control (Millipore) in RIP buffer. RNA in immunoprecipitate was separated with TRIzol reagent and analyzed by qRT-PCR.

### Dual-Luciferase Assay

For dual-luciferase assay on VPS9D1-AS1 and miRNA-30a-5p in this study, the amplified VPS9D1-AS1 mutant (VPS9D1-AS1-Mut) or wild-type (VPS9D1-AS1-Wt) 3′UTR was cloned into the multiple cloning sites of the downstream of pmirGLO (Promega, Madison, USA) luciferase reporter vector. Lipofectamine 2000 was used to co-transfect VPS9D1-AS1-Mut or VPS9D1-AS1-Wt vector with miRNA-30a-5p-mimic or NC-mimic into the cells.

For dual-luciferase assay on miRNA-30a-5p and KIF11, the amplified KIF1-Mut or KIF11-Wt 3’ UTR was cloned into multiple cloning sites of the downstream of pmirGLO (Promega, Madison, USA) luciferase reporter vector. Lipofectamine 2000 was utilized to co-transfect KIF11-Mut or KIF11-Wt vector with miRNA-30a-5p-mimic or NC-mimic vector into the cells. After 48 h of transfection, luciferase reporter analysis system (Promega) was introduced to assay firefly and renilla luciferase activities.

### Statistical Analysis

All data were processed with GraphPad Prism 7 software (GraphPad Software, Inc., CA). All experiments were carried out three times in both technical repetition and biological repetition. Measurement data were presented as mean ± SD. Comparison between the two groups was determined by *t*-test. ANOVA was used for comparison among multiple groups. *p* < 0.05 means a statistically significant difference.

## Results

### VPS9D1-AS1 Is Upregulated in LUAD

**FIGURE 1:**
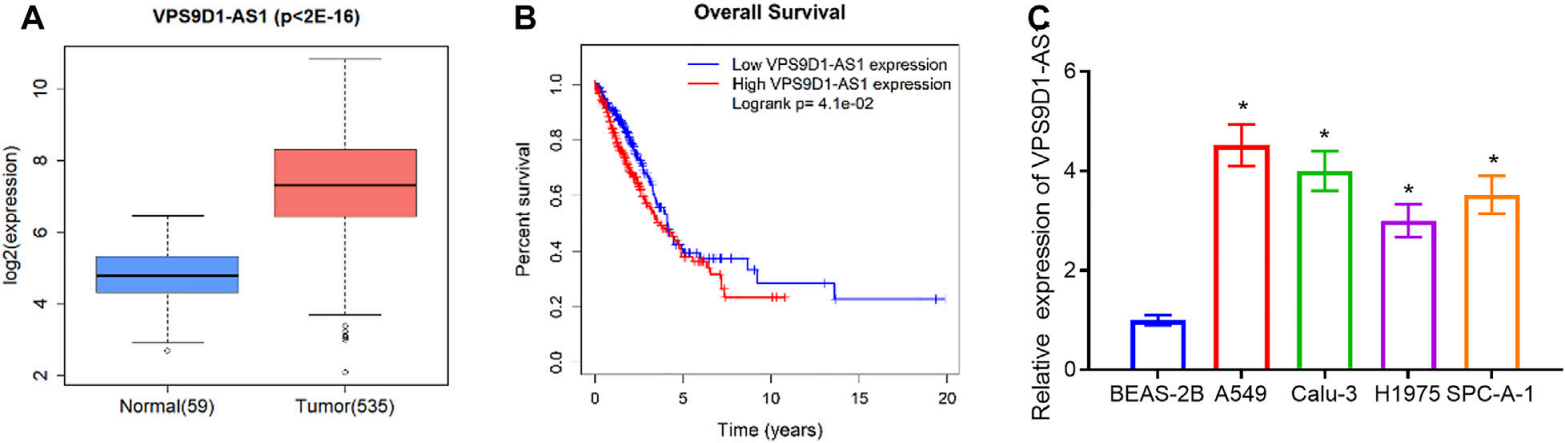
VPS9D1-AS1 is increased in LUAD. **(A)** Box plot of VPS9D1-AS1 level. Blue box, normal tissue; red box, LUAD tissue. **(B)** Survival analysis of high- and low-expression groups with the median value of VPS9D1-AS1 as the cutoff value. **(C)** qRT-PCR assayed VPS9D1-AS1 level in BEAS-2B and A549, Calu-3, H1975, and SPC-A-1 cells. * represents *p* < 0.05 in comparison with BEAS-2B group. All assays were repeated three times in triplicate.

Some literature manifested that VPS9D1-AS1 is notably highly expressed in varying cancer types and can regulate occurrence and progression of cancer (Yang et al., 2016; Tan and Yang, 2018; Han et al., 2020; Hou et al., 2020). However, the regulatory mechanism of VPS9D1-AS1 in LUAD is still unclear. Hence, we chose VPS9D1-AS1 as the object in this research. We tested VPS9D1-AS1 level in normal tissue and LUAD tissue in TCGA database by *t*-test. VPS9D1-AS1 was proven remarkably increased in LUAD tissue (Figure 1A). High VPS9D1-AS1 level was correlated with unfavorable prognosis (Figure 1B). However, there was no significant correlation between the abnormal expression of VPS9D1-AS1 and clinical pathology (Supplementary Figure S1). Similarly, we conducted qRT-PCR to detect VPS9D1-AS1 level in BEAS-2B and A549, Calu-3, H1975, and SPC-A-1 cells. VPS9D1-AS1 was increased in all tested LUAD cells (Figure 1C). Through these results, we finally determined that VPS9D1-AS1 was increased in LUAD.

### VPS9D1 Facilitates the Cell Proliferation, Migration, and Invasion of LUAD Cells

**FIGURE 2:**
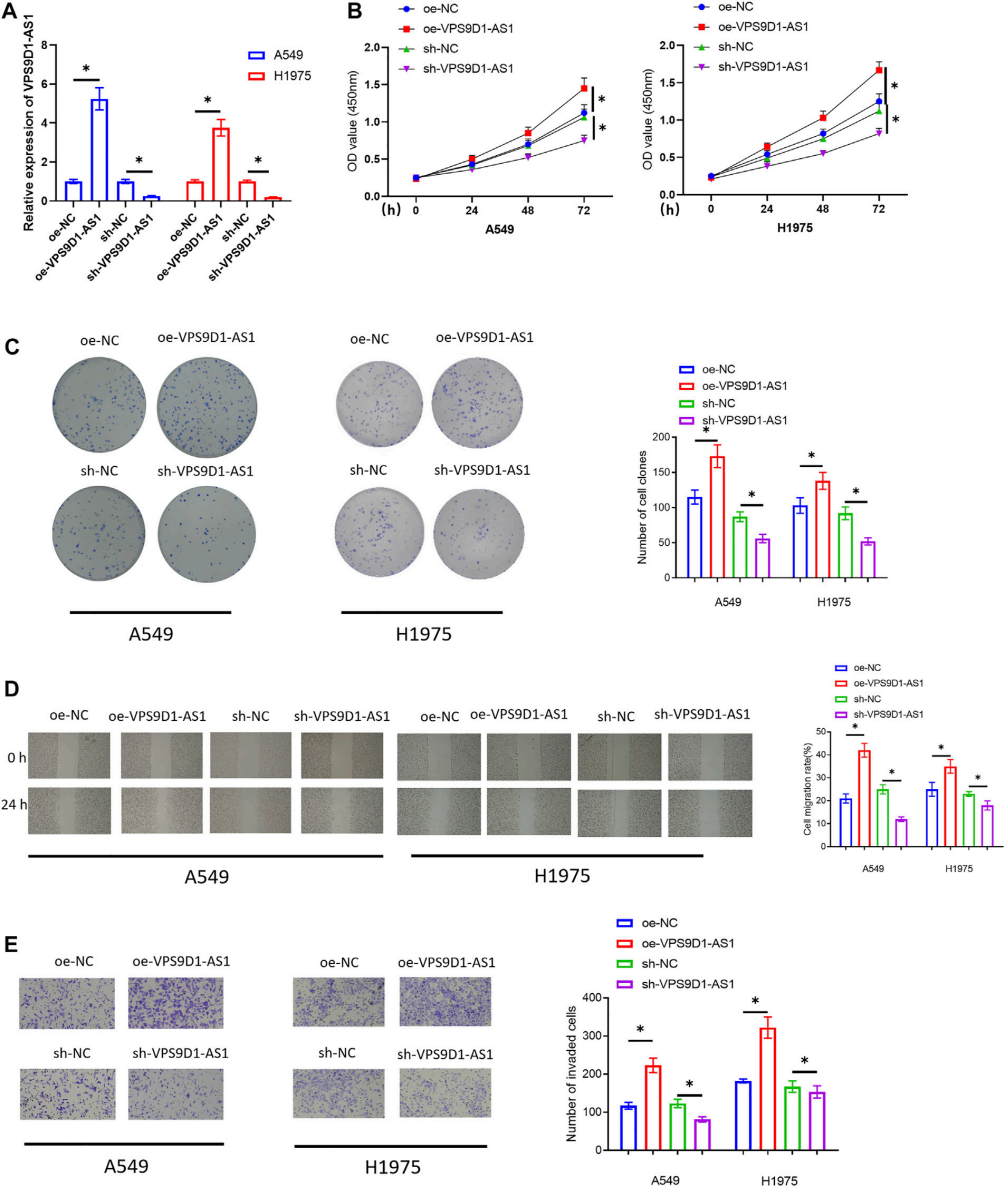
VPS9D1-AS1 facilitates the cell proliferation, migration, and invasion of LUAD. **(A)** The effect of transfecting oe-VPS9D1-AS1 and sh-VPS9D1-AS1 on VPS9D1-AS1 expression in A549 and H1975 cells. **(B)** The impact of overexpressing or silencing VPS9D1-AS1 on the viability of the LUAD cells. **(C)** The impact of overexpressing or silencing VPS9D1-AS1 on colony formation ability of the LUAD cells. **(D)** The impact of overexpressing or silencing VPS9D1-AS1 on migratory ability of the LUAD cells (40×). **(E)** The influence of overexpressing or silencing VPS9D1-AS1 on invasive ability of the LUAD cells (100 ×). * represents *p* < 0.05 in comparison with the negative control group. All assays were repeated three times in triplicate.

To unveil how VPS9D1 functioned in LUAD progression, A549 and H1975 cells were chosen for the following related assays. First, we transfected oe-VPS9D1-AS1 and sh-VPS9D1-AS1 into the cells to construct the VPS9D1-AS1 overexpression or silence cell models. The overexpression and silence efficiencies were observed by qRT-PCR (Figure 2A). Then, cellular biological experiments were conducted to observe the impact of overexpressing or silencing VPS9D1-AS1 on the proliferation, migration, and invasion of the cells. The results of CCK-8 assay and colony formation assay indicated that overexpressing VPS9D1-AS1 could notably facilitate LUAD cell proliferation, whereas silencing VPS9D1-AS1 inhibited the cell proliferation (Figures 2B,C). In results of scratch healing assay and cell invasion assay, we also observed that overexpressing VPS9D1-AS1 fostered migration and invasion of the LUAD cells, whereas knockdown VPS9D1-AS1 repressed cell migration and invasion (Figures 2D,E). Through cellular biological function experiments, we fully confirmed that VPS9D1-AS1 could promote the malignant progression of LUAD cells.

### VPS9D1-AS1 Can Be a Molecular Sponge for miRNA-30a-5p

**FIGURE 3:**
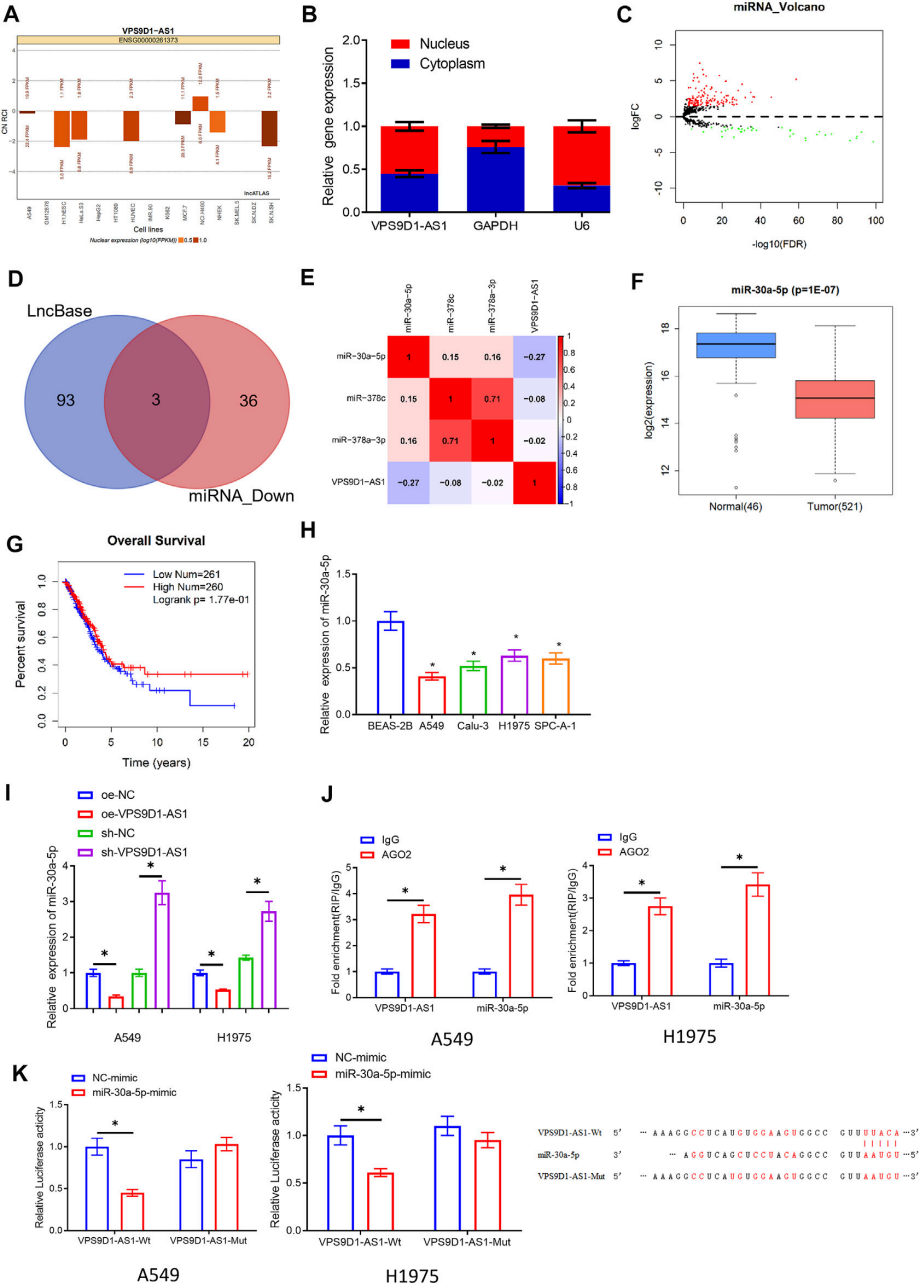
VPS9D1-AS1 can be a molecular sponge for miRNA-30a-5p. **(A)** LncATLAS database was used for subcellular localization analysis of VPS9D1-AS1. **(B)** Subcellular fractionation and qRT-PCR were introduced to analyze relative expression of VPS9D1-AS1 in the nuclear and cytoplasmic of A549 cells. **(C)** Volcano plot of differentially expressed miRNAs in TCGA-LUAD. Red indicates increased miRNAs, and green represents decreased miRNAs. **(D)** Intersection of miRNAs that have binding sites with VPS9D1-AS1 and differentially downregulated miRNAs in LUAD in LncBase database. **(E)** The correlation of VPS9D1-AS1, miRNA-30a-5p, miRNA-308c, and miRNA-378a-3p levels in LUAD tissue. **(F)** Box plot of miRNA-30a-5p level. Blue plot indicates normal tissue, and red plot indicates LUAD tissue. **(G)** Survival analysis of high- and low-expression groups with the median value of miRNA-30a-5p as the cutoff value. **(H)** Expression of miRNA-30a-5p in BEAS-2B and A549, Calu-3, H1975, and SPC-A-1 cells (* represents *p* < 0.05 in comparison with BEAS-2B group). **(I)** Effect of overexpressing or silencing VPS9D1-AS1 on miRNA-30a-5p level in A549 and H1975 cells (* represents *p* < 0.05 in comparison with the negative control group). **(J)** Interaction between VPS9D1-AS1 and miRNA-30a-5p. IgG was a negative control (* represents *p* < 0.05 in comparison with IgG group). **(K)** Binding of VPS9D1-AS1 and miRNA-30a-5p (* represents *p* < 0.05 in comparison with the negative control group). All assays were repeated three times in triplicate.

We mined the miRNAs related to VPS9D1-AS1 through bioinformatics on the basis of ceRNA hypothesis. First, we used lncATLAS database and subcellular fractionation to observe the subcellular location of VPS9D1-AS1. VPS9D1-AS1 was expressed both in cytoplasm and the nucleus (Figures 3A,B), demonstrating that VPS9D1-AS1 could play a modulatory role as a ceRNA in LUAD. Then, we predicted the interacting miRNAs of VPS9D1-AS1 by LncBase. The predicted miRNAs were intersected with the 39 differentially downregulated miRNAs, and three miRNAs were obtained: miRNA-30a-5p, miRNA-378a-3p, and miRNA-378c (Figures 3C,D). Later, we performed Pearson correlation analysis between the predicted miRNAs and VPS9D1-AS1. MiRNA-30a-5p had the highest inverse correlation, which was selected for the research (Figure 3E). In TCGA-LUAD database, downregulation of miRNA-30a-5p in LUAD was shown in Figure 3F. Survival analysis unveiled that prognosis of patients with low miRNA-30a-5p level was better than that of patients with high level (Figure 3G). To verify the above results, qRT-PCR was performed on BEAS-2B and A549, Calu-3, H1975, and SPC-A-1 cells, which suggested that miRNA-30a-5p was conspicuously increased in LUAD cells (Figure 3H). In addition, qRT-PCR was utilized to observe miRNA-30a-5p level in A549 and H1975 cells with overexpressed or silenced VPS9D1-AS1. The results indicated that overexpressing VPS9D1-AS1 reduced miRNA-30a-5p level, whereas knockdown miRNA-30a-5p enhanced miRNA-30a-5p (Figure 3I). Through these results, we proved that miRNA-30a-5p and VPS9D1-AS1 expression was negatively correlated in LUAD. Next, we observed through RIP that AGO2 could be enriched in VPS9D1-AS1 and miRNA-30a-5p in the cells (Figure 3J), which showed that miRNA-30a-5p and VPS9D1-AS1 could interact with each other. At the same time, in dual-luciferase assay, we also noticed that miRNA-30a-5p-mimic did not affect luciferase activity of VPS9D1-AS1-Mut but reduced that of VPS9D1-AS1-Wt. This result manifested that there were binding sites of miRNA-30a-5p and VPS9D1-AS1 (Figure 3K). In general, these data fully confirmed that VPS9D1-AS1 directly interacted with miRNA-30a-5p.

### MiRNA-30a-5p Restores the Function of VPS9D1-AS1 on Promoting Cell Proliferation, Migration, and Invasion of LUAD

**FIGURE 4:**
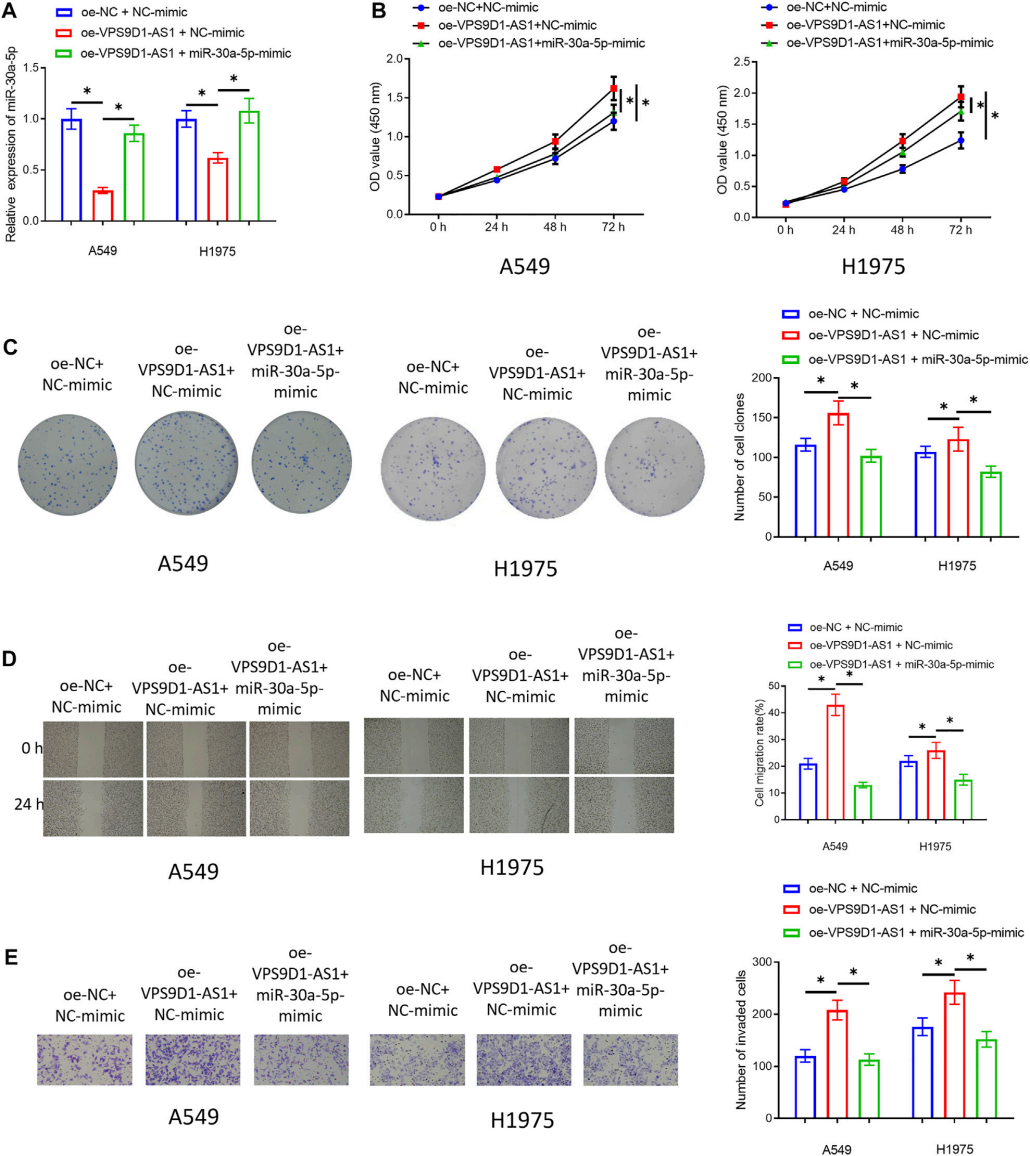
MiRNA-30a-5p restores the effect of VPS9D1 on promoting cell proliferation, migration, and invasion of LUAD. **(A)** Overexpression efficiency of transfecting miRNA-30a-5p-mimic on miRNA-30a-5p level in A549 and H1975 cells. **(B)** The impact of simultaneous overexpression of VPS9D1-AS1 and miRNA-30a-5p on the viability of the LUAD cells. **(C)** Effect of simultaneous overexpression of VPS9D1-AS1 and miRNA-30a-5p on the colony formation ability of the LUAD cells. **(D)** The impact of simultaneous overexpression of VPS9D1-AS1 and miRNA-30a-5p on migratory ability of the LUAD cells (40 ×). **(E)** The impact of simultaneous overexpression of VPS9D1-AS1 and miRNA-30a-5p on invasive ability of the LUAD cells (100 ×). * represents *p* < 0.05 when oe-NC + NC-mimic group was compared to oe-VPS9D1-AS1 + NC-mimic group and when oe-VPS9D1-AS1 + NC-mimic group was compared to oe-VPS9D1-AS1+miR-30a-5p-mimic group. All assays were repeated three times in triplicate.

In *VPS9D1-AS1 Can Be a Molecular Sponge for miRNA-30a-5p*, we confirmed that VPS9D1-AS1 could sponge miRNA-30a-5p, thereby inhibiting miRNA-30a-5p, but whether miRNA-30a-5p can regulate malignant progression of LUAD was still unknown. Therefore, we co-transfected oe-VPS9D1-AS1 and miRNA-30a-5p-mimic in A549 and H1975 cells and constructed the three transfected groups (oe-NC + NC-mimic group, oe-VPS9D1-AS1 + NC-mimic group, and oe-VPS9D1-AS1 + miRNA-30a-5p-mimic group). qRT-PCR was used to verify that simultaneous transfection of miRNA-30a-5p-mimic could restore miRNA-30a-5p level in the cells to a certain extent (Figure 4A). Then, cellular experiments checked the impact of simultaneous transfection of miRNA-30a-5p-mimic on proliferation, migration, and invasion of these cells. The results suggested that overexpressing miRNA-30a-5p could significantly restore the promoting impact of VPS9D1-AS1 on malignant behaviors of LUAD cells (Figures 4B–E). Hence, VPS9D1-AS1 could accelerate malignant progression of LUAD *via* sponging miRNA-30a-5p.

### VPS9D1-AS1 Upregulates KIF11 Expression Through Competitive Sponging miRNA-30a-5p

**FIGURE 5:**
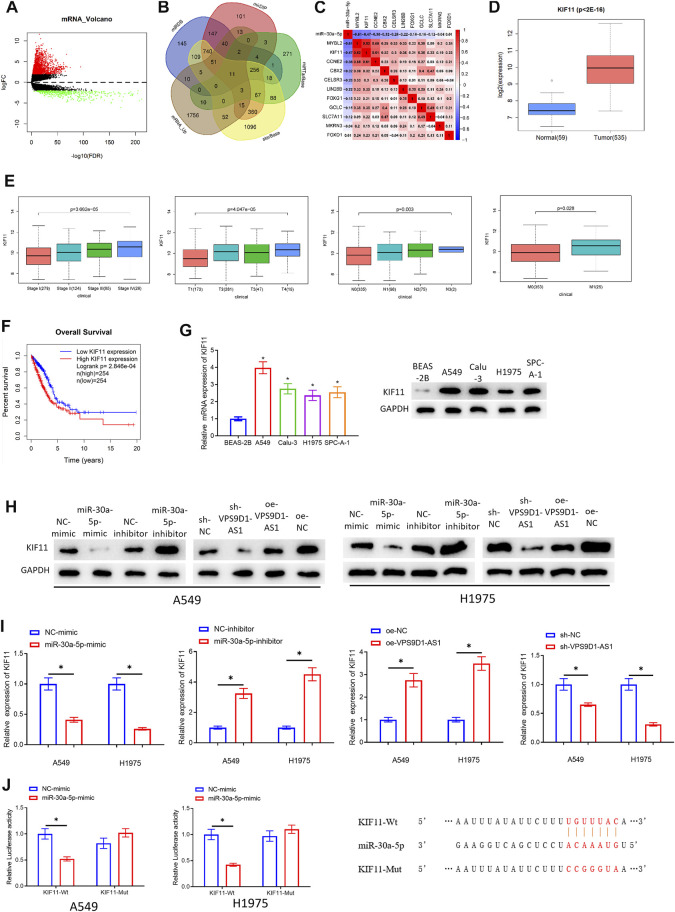
VPS9D1-AS1 upregulates KIF11 expression through competitive adsorption of miRNA-30a-5p. **(A)** Volcano plot of differentially expressed mRNAs in TCGA-LUAD. Red, increased mRNAs; green, decreased miRNAs. **(B)** The mRNAs that had binding sites with miRNA-30a-5p identified by miRDB, mirDIP, miRTarBase, and starBase databases and were overlapped with the differentially increased mRNAs in LUAD. **(C)** The correlation between MYBL2, KIF11, CCNE2, CBX2, CELSR3, LIN28B, FOXG1, GCLC, SLC7A11, MKRN3, FOXD1, and miRNA-30a-5p in LUAD tissue. **(D)** Box plot of KIF11 level. Blue indicates normal tissue, and red indicates LUAD tissue. **(E)** Correlation between KIF11 and clinical features (Stage, T, M, and N). **(F)** Survival curve of KIF11. The abscissa, time (in years); the ordinate, survival rate. Red curve, high expression; blue curve, low expression. **(G)** KIF11 level in BEAS-2B and A549, Calu-3, H1975, and SPC-A-1 cells (* represents *p* < 0.05 in comparison with BEAS-2B group). **(H, I)** Effects of overexpressing or silencing miRNA-30a-5p and overexpressing or silencing VPS9D1-AS1 on the protein and mRNA expression of KIF11 in A549 and H1975 cells (* represents *p* < 0.05 in comparison with the negative control group). **(J)** The targeting relationship of miRNA-30a-5p and KIF11 (* represents *p* < 0.05 in comparison with BEAS-2B group). All assays were repeated three times in triplicate.

To further study regulatory mechanism of VPS9D1-AS1, it is necessary to mine the downstream mRNAs regulated by miRNA-30a-5p. MRNAs that had binding sites with miRNA-30a-5p were predicted through bioinformatics analysis. The predicted mRNAs were intersected with the 1.968 upregulated mRNAs. Finally, 11 target genes (MYBL2, KIF11, CCNE2, CBX2, CELSR3, LIN28B, FOXG1, GCLC, SLC7A11, MKRN3, and FOXD1) were obtained (Figures 5A,B). The correlation between the 11 target mRNAs and miRNA-30a-5p was analyzed (Figure 5C). The FDR values of these 11 target mRNAs were observed (Supplementary Table S1). Differential analysis was done on the gene expression data between normal and cancer group, and the research progress on these mRNAs in LUAD was checked (Li et al., 2020a; Xiong et al., 2020). We finally selected KIF11 as the research object. The upregulated KIF11 in LUAD in TCGA-LUAD database was shown in Figure 5D. In addition, Wilcoxon test (2 groups) or Kruskal test (>2 groups) was used to test the correlation between KIF11 and clinical features. We also disclosed that KIF11 level was notably positively related to clinical features (Stage, T, M, and N) (Figure 5E). Survival analysis indicated that patients with LUAD with increased KIF11 had an unfavorable prognosis than those with decreased KIF11 (Figure 5F). To verify that KIF11 was the downstream target of miRNA-30a-5p, qRT-PCR and Western blot assays were performed to observe the mRNA and protein levels of KIF11 in BEAS-2B and A549, Calu-3, H1975, and SPC-A-1 cells. From the results, we verified that KIF11 was increased in LUAD cells (Figure 5G). At the same time, the results of qRT-PCR and Western blot assay denoted that overexpressing miRNA-30a-5p or silencing VPS9D1-AS1 could noticeably downregulate KIF11 level. Adversely, silencing miRNA-30a-5p or overexpressing VPS9D1-AS1 could upregulate the expression of KIF11 (Figures 5H,I). Finally, dual-luciferase assay also manifested that miRNA-30a-5p-mimic was irrelevant to luciferase activity of KIF11-Mut, but it would reduce that of KIF11-Wt (Figure 5J). Through these assays, we fully verified that VPS9D1-AS1 could increase KIF11 level *via* competitive sponging miRNA-30a-5p in LUAD cells.

### KIF11 Can Restore the Impact of miRNA-30a-5p on Repressing the Cell Proliferation, Migration, and Invasion of LUAD

**FIGURE 6:**
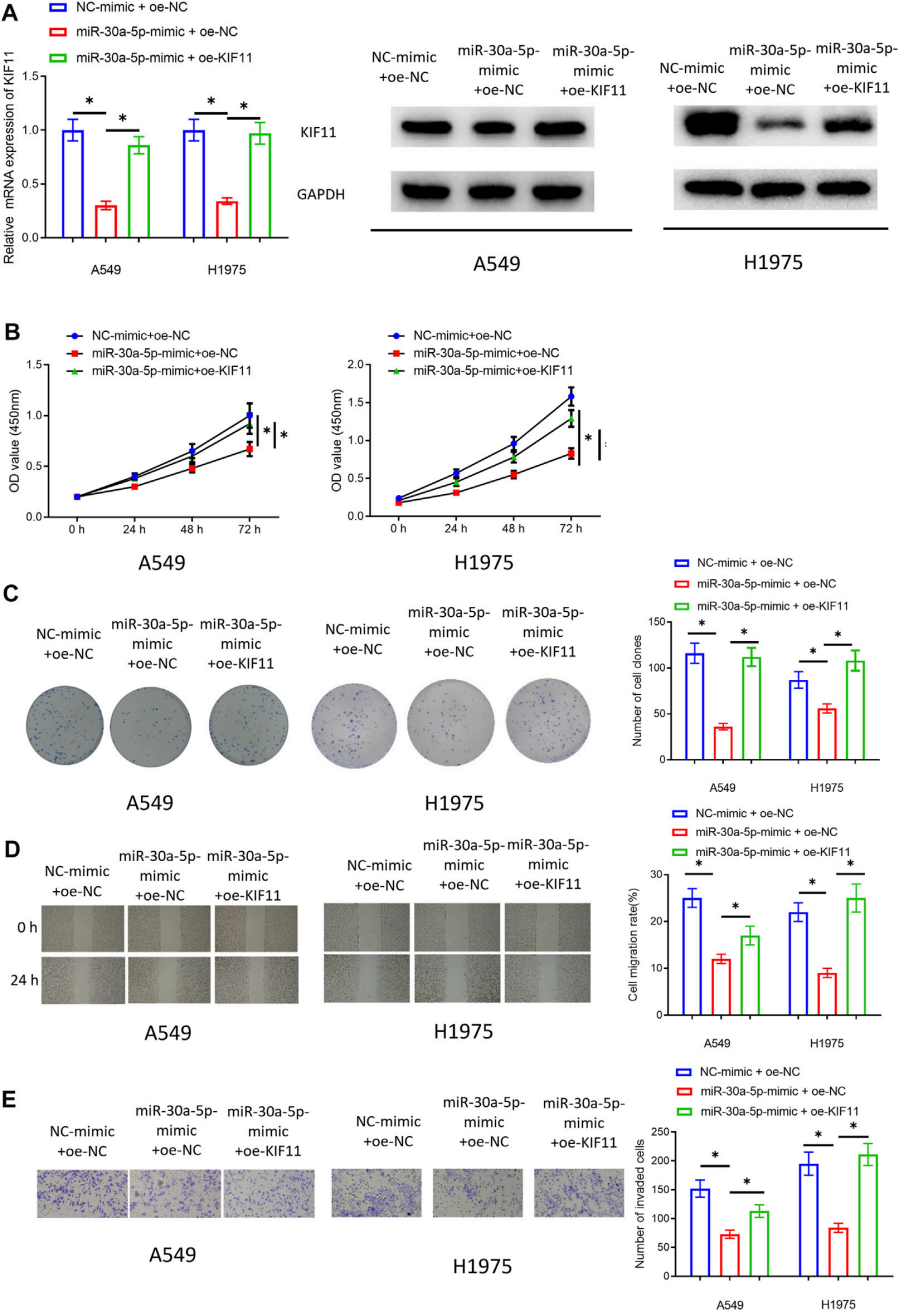
KIF11 can restore the inhibitory impact of miRNA-30a-5p on cell proliferation, migration, and invasion of LUAD. **(A)** Effect of KIF11 expression in A549 and H1975 cells. **(B)** The impact of simultaneously overexpressing miRNA-30a-5p and KIF11 on the viability of LUAD cells. **(C)** The impact of simultaneously overexpressing miRNA-30a-5p and KIF11 on colony formation ability of LUAD cells. **(D)** The influence of simultaneously overexpressing miRNA-30a-5p and KIF11 on migratory ability of LUAD cells (40 ×). **(E)** The effect of simultaneously forced expression of miRNA-30a-5p and KIF11 on invasive property of LUAD cells (100 ×). * represents *p* < 0.05 when NC-mimic + oe-NC group was compared to miR-30a-5p-mimic + oe-NC group and when miR-30a-5p-mimic + oe-NC was compared to miR-30a-5p-mimic + oe-KIF11 group. All assays were repeated three times in triplicate.

In *VPS9D1-AS1 Upregulates KIF11 Expression Through Competitive Sponging miRNA-30a-5p*, we used bioinformatics to find that KIF11 and clinical features (Stage, T, M, and N) were significantly positively correlated, and KIF11 might be an unfavorable prognostic factor for patients with LUAD. Therefore, by performing rescue experiments, we subsequently observed whether KIF11 could restore the inhibitory impact of miRNA-30a-5p on malignant progression of LUAD cells, and the bioinformatics results were also verified. We simultaneously transfected miRNA-30a-5p-mimic and oe-KIF11 in the LUAD cells. Cells were divided into NC-mimic + oe-NC group, miRNA-30a-5p-mimic + oe-NC group, and miRNA-30a-5p-mimic + oe-KIF11 group. qRT-PCR and Western blot assay were performed to validate that transfecting oe-KIF11 could remarkably increase KIF11 level (Figure 6A). Then, cellular function experiments were conducted to assay proliferation, migration, and invasion of the cells. The results suggested that KIF11 could indeed rescue inhibitory impact of miRNA-30a-5p in LUAD cells (Figures 6B–E). Hence, we fully proved that miRNA-30a-5p could inhibit malignant phenotypes of LUAD cells through KIF11.

## Discussion

LncRNAs were considered as non-functional “junk sequences” and “transcriptional noise,” but increasing evidence confirmed that many lncRNAs can modulate occurrence and progression of cancers (Loewer et al., 2010; Liu et al., 2018; Luo et al., 2018). Therefore, in this study, we selected VPS9D1-AS1, with an unclear specific regulatory mechanism in LUAD, as the research object. VPS9D1-AS1 was proven overexpressed in lung cancer cells and tissue (Wang et al., 2019a). Similarly, in our study, it was confirmed that VPS9D1-AS1 was upregulated in LUAD. LUAD progress was facilitated *via* VPS9D1-AS1/miRNA-30a-5p/KIF11 axis. To sum up, highly expressed VPS9D1-AS1 was positively related to poor prognosis of patients with LUAD. VPS9D1-AS1 was first proven that it could facilitate the proliferation, migration, and invasion of LUAD cells. It could be a pro-oncogenic factor in LUAD.

MiRNAs are endogenous non-coding RNAs (Sontheimer and Carthew, 2005). lncRNA was confirmed to play a regulatory role in LUAD through sponge of miRNA. For instance, TTN-AS1 induces proliferation and migration of LUAD cells *via* sponge of miRNA-4677-3p (Zhong et al., 2019). MAFG-AS1 can hasten proliferation of LUAD cells *via* sponge of miRNA-774-5p (Sui et al., 2019). Therefore, we observed subcellular localization of VPS9D1-AS1 through lncATLAS database and subcellular fractionation. VPS9D1-AS1 was proven expressed both in cytoplasm and nucleus. Subsequently, miRNAs that could be sponged by VPS9D1-AS1 were excavated, and miRNA-30a-5p, miRNA-378a-3p, and miRNA-378c were finally obtained. Because the inverse correlation between miRNA-30a-5p and VPS9D1-AS1 was the highest, miRNA-30a-5p was selected for study. Meanwhile, research reported that miRNA-30a-5p can be sponged by a variety of lncRNAs, including XIXT, DLEU2, FEZF1-AS1, MALAT1, and NORAD (Pan et al., 2018; Zhang et al., 2019; Li et al., 2020b; Li et al., 2020c; Wu et al., 2020). In addition, studies indicated that miRNA-30a-5p can repress progression of a variety of cancers, like ovarian cancer (Wang et al., 2019b), oral cancer (Ruan et al., 2018), colorectal cancer (Wei et al., 2016), osteosarcoma (Tao et al., 2018), and cutaneous squamous cell carcinoma (Shao et al., 2019). We determined that miRNA-30a-5p was noticeably decreased in LUAD cells. VPS9D1-AS1 was confirmed sponging miRNA-30a-5p. It was also confirmed that miRNA-30a-5p restored the impact of VPS9D1-AS1 on promoting malignant phenotypes of LUAD cells. Thus, we fully verified that VPS9D1-AS1 could induce malignant progression of LUAD through sponge of miRNA-30a-5p.

More and more studies showed that miRNA can induce mRNA degradation or inhibit protein translation, thereby modulating cancer progression (Fang et al., 2015; Enokida et al., 2016). We used bioinformatics to mine the downstream mRNAs of miRNA-30a-5p, and a total of 11 target genes were obtained, namely, MYBL2, KIF11, CCNE2, CBX2, CELSR3, LIN28B, FOXG1, GCLC, SLC7A11, MKRN3, and FOXD1. By observing the correlation between the 11 target mRNAs and miRNA-30a-5p, The FDR values of the 11 target mRNAs, as well as the research progress of them in LUAD, KIF11 was selected for research. KIF11 is a member of the KIF family. Like other KIF members, it has important functions in cells, which is involved in mitosis, and transportation of intracellular vesicles and organelles (Hirokawa, 1998; Lawrence et al., 2004; Wordeman, 2010; Hirokawa and Tanaka, 2015). The latest research suggested that KIF11 also exerts an essential regulatory effect on cancer progression. For example, Bianhua Shi reported that KIF11 can promote the migration of ovarian cancer cells (Shi et al., 2018). Kayo Daigo found that downregulating KIF11 can hasten apoptosis of oral cancer cells, inhibiting cell proliferation at the same time (Daigo et al., 2018). However, it is not known yet whether KIF11 can modulate LUAD malignant progression. We determined that KIF11 was noticeably increased in LUAD cells and confirmed that KIF11 was a downstream target of miRNA-30a-5p. Upregulating KIF11 level restored the inhibitive impact of miRNA-30a-5p on the proliferation, migration, and invasion of LUAD cells. The data fully indicated that miRNA-30a-5p inhibited the malignant phenotype of LUAD cells by targeting KIF11.

In summary, in this study, we found that VPS9D1-AS1 was a tumor-promoting factor for LUAD, which could target KIF11through sponge adsorption of miRNA-30a-5p to promote the malignant progression of LUAD. This discovery revealed a novel regulatory axis of VPS9D1-AS1/miRNA-30a-5p/KIF11 that regulated LUAD cell progression. However, these results are subject to certain limitations. We only verified the results at cellular level. Clinical tissue should be collected for further *in vivo* experiments. Thus, evidence together lie more theoretical groundworks that VPS9D1-AS1 is a possible target for LUAD treatment.

## Data Availability

The original contributions presented in the study are included in the article/Supplementary Material. Further inquiries can be directed to the corresponding author.

## Supplementary Material

Supplementary Figure 1 The correlation between the expression of lncRNA VPS9D1 and clinical pathology of patients. **(A)** The correlation between the expression of VPS9D1 and T stage. **(B)** The correlation between VPS9D1 expression and N stage. **(C)** The correlation between VPS9D1 expression and M stage. **(D)** The correlation between VPS9D1 expression and clinical stage. ns represents no statistically significance of difference.
