# Supplementary figures and images for: LncRNA VPS9D1-AS1 Promotes Malignant Progression of Lung Adenocarcinoma by Targeting miRNA-30a-5p/KIF11 Axis
Source: Front Genet. 2022 Jan 24;12:807628. doi: 10.3389/fgene.2021.807628 (PMC8819668; doi:10.3389/fgene.2021.807628)

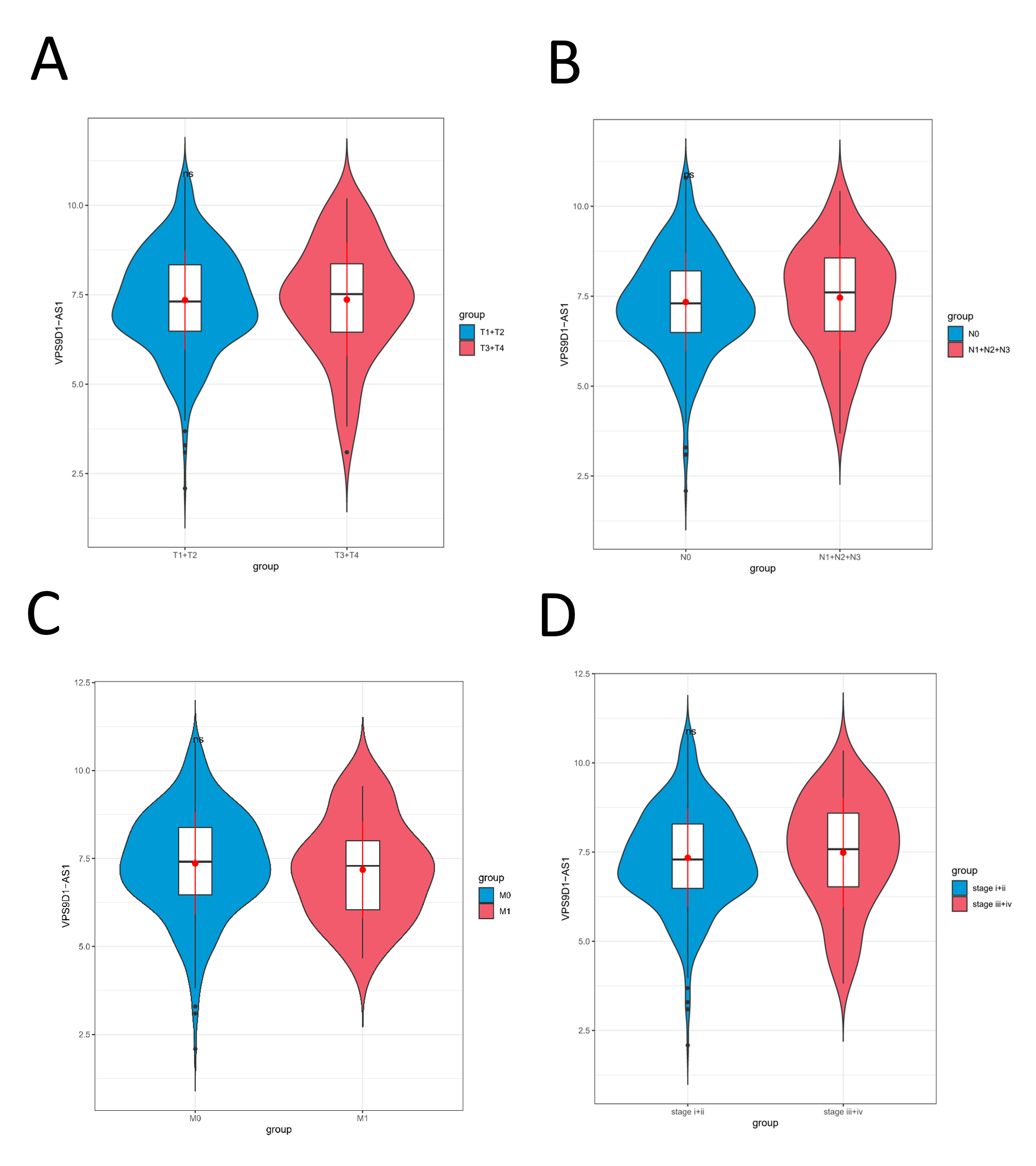

Supplement: Supplementary file 1 [file Image1.tif]
